# An Overview of the Health Benefits, Extraction Methods and Improving the Properties of Pomegranate

**DOI:** 10.3390/antiox12071351

**Published:** 2023-06-27

**Authors:** Giada Benedetti, Federica Zabini, Luca Tagliavento, Francesco Meneguzzo, Vincenzo Calderone, Lara Testai

**Affiliations:** 1 Department of Pharmacy, University of Pisa, Via Bonanno 6, 56120 Pisa, Italy; g.benedetti18@studenti.unipi.it (G.B.); vincenzo.calderone@unipi.it (V.C.); 2 Istituto per la Bioeconomia, CNR, Via Madonna del Piano 10, 50019 Sesto Fiorentino, Italy; federica.zabini@ibe.cnr.it; 3 HyRes Srl, Via Salvator Rosa 18, 82100 Benevento, Italy; luca.tagliavento@hyres.it; 4 Interdeparmental Center of Nutrafood, University of Pisa, Via del Borghetto, 56120 Pisa, Italy

**Keywords:** *Punica granatum*, nutraceuticals, ellagitannins, by-products, green chemistry

## Abstract

Pomegranate (*Punica granatum* L.) is a polyphenol-rich edible food and medicinal plant of ancient origin, containing flavonols, anthocyanins, and tannins, with ellagitannins as the most abundant polyphenols. In the last decades, its consumption and scientific interest increased, due to its multiple beneficial effects. Pomegranate is a balausta fruit, a large berry surrounded by a thick colored peel composed of exocarp and mesocarp with edible arils inside, from which the pomegranate juice can be produced by pressing. Seeds are used to obtain the seed oil, rich in fatty acids. The non-edible part of the fruit, the peel, although generally disposed as a waste or transformed into compost or biogas, is also used to extract bioactive products. This review summarizes some recent preclinical and clinical studies on pomegranate, which highlight promising beneficial effects in several fields. Although further insight is needed on key aspects, including the limited oral bioavailability and the role of possible active metabolites, the ongoing development of suitable encapsulation and green extraction techniques enabling the valorization of waste pomegranate products point to the great potential of pomegranate and its bioactive constituents as dietary supplements or adjuvants in therapies of cardiovascular and non-cardiovascular diseases.

## 1. Introduction

Pomegranate (*Punica granatum*) is a fruit-bearing shrub that belongs to the Punicaceae family, arising interest worldwide in terms of cultural, traditional and therapeutic usages; it is known as the *fruit of Eden* for its delicious taste and health benefiting properties [1,2,3]. This plant is native to Iran and Afghanistan, and nowadays, its cultivation is also spread in Africa, America, the Middle East, Australia and Europe, and is consumed as a fresh fruit or beverage after processing it into a juice, jam, oil or infusion [4,5].

Pomegranate is a balausta fruit, a large berry surrounded by a thick colored peel composed by exocarp and mesocarp. The white mesocarp forms chambers (locules) containing edible arils with seeds inside. From the red arils, which account for 45–52% of the total fruit weight, the pomegranate juice (PJ) can be produced. Seeds represent 12–20% of the fruit and are used to obtain the seed oil, rich in unsaturated fatty acids. Finally, the non-edible part of the fruit is the peel (40–55%), which is generally regarded as a waste and disposed or conveyed to compost or biogas generation plants. However, pomegranate peel has recently come of interest for the extraction of bioactive compounds. Indeed, in recent years numerous techniques have been set up to exploit pomegranate peel or seeds and extract their active components [6,7].

The composition of the phytochemicals in pomegranate depends on the cultivation, the geographic region, the climate conditions, the varieties and the extraction method [8]. Kumar et al., for example, compared the levels of phenolic compounds between two pomegranate varieties and the results showed that in *bhagwa* pomegranate the quantity of phytochemicals was markedly higher than the *ganesh* variety [9]; moreover, pomegranate grown in the desert climate showed higher levels of phenolic compounds and lower content of organic acids compared to the ones grown in a Mediterranean climate [10,11,12].

Pomegranate composition is also different in its distinct parts (arils, seeds, peel in the fruit, along with flowers, leaves, bark), with more than 500 compounds identified through various analytical techniques, such as liquid chromatography—mass spectrometry with high resolution, by using diode array detection, electron spin resonance, fluorescence detection, flame ionization detection, infrared spectroscopy, mass spectrometry, nuclear magnetic resonance and thin layer chromatography [13,14].

The bioactive constituents of pomegranate include primary metabolites, such as sugars, lipides and fatty acids, as well as secondary metabolites, such as tannins, organic acids, phenolic acids (including gallic acid, ellagic acid (EA), caffeic acid), anthocyanins, flavonoids, vitamins and terpenes [12]. However, the main secondary metabolites seem to be tannins, including ellagitannins and gallotannins [4,14].

Based on these interesting prospectives in nutraceutical field, we summarized, using PubMed and Google Scholar databases, the most recent preclinical and clinical studies on pomegranate, highlighting promising beneficial effects in several fields. Biopharmaceutical studies addressed to improve the oral bioavailability of active metabolites, as well as the efforts to use green extraction techniques enabling the sustainable exploitation of waste and by-products of the pomegranate supply chain, have been analyzed, as they are considered critical aspects for the clinical use as dietary supplements or adjuvants in therapies of cardiovascular and non-cardiovascular diseases.

## 2. Phytochemical Composition of Different Parts of the Plant

### 2.1. Fruits

Arils are the edible part of pomegranate, made of a red pulp (78%) and seeds (22%); they are used to squeeze the PJ and are endowed with a sweet–sour taste [6,15]. Arils are composed of 85% water, 10% sugars and 5% of other bioactive compounds [12,16]. Numerous studies, using different pomegranate varieties, indicate that glucose and fructose are the main sugars present in arils [12,17,18]. Glutamine, serine, aspartate and alanine are the dominant amino acids [19]. Moreover, arils contain hydrolyzed tannins and, in particular, ellagitannins, such as punicalagins, punicalin, gallic acid, corilagin and EA [20,21], polyphenols, such as flavonoids (catechin, epicatechin, quercetin, rutin, epigallocatechin), and anthocyanins, which are responsible for a brilliant red color [20,22]. Anthocyanin content shifts from 9 to 115 mg/L juice [18], including compounds like delphinidin, cyanidin and pelargonidin [23].

Arils are rich even in organic acids, such as citric acid, malic acid, succinic acid and oxalic acid, that are responsible for the acid–sour taste [12,18], as well as in vitamin C, a powerful antioxidant, in a quantity similar to Citrus fruits [24,25]. Finally, arils are a source of minerals, for example, magnesium, boron, selenium, zinc, cobalt, calcium and sodium in different amounts depending on cultivars and varieties [22,26].

An innovative product is fermented juice, obtained through fermentation with different kinds of yeasts and characterized by an enrichment of bioactive compounds, in particular of free polyphenols, due to the ability of the fermentation process to break flavonoid–sugar linkages. Kim et al. described a process characterized by a fermentation with wine yeast (*Saccharomyces bayanus*, Prise de Mousse) for 2 months. After alcohol remotion, the liquor was concentrated and added with ethyl acetate, and then, it was dried in a vacuum evaporator to obtain the residue [27]. On the other hand, Akter et al. described the production of a fermented juice using *Lactobacillus vespulae* DCY75 yeast and tannin acyl hydrolase. Through their synergistic action, tannins were converted into EA, increasing its concentration up to 70%. In particular, comparing the fermented and non-fermented PJ, the fermentation process increased the amount of flavonoids and phenol compounds, resulting in enhanced biological activity [28].

### 2.2. Seeds

Seeds represent an inedible fraction of pomegranate, correspond to 11% *w/w* of the fruit and contain the largest pomegranate lipidic fraction; they are mainly used to extract the pomegranate seeds oil (PSO) [12,29,30,31,32].

PSO is rich in fatty acids, which are mostly poly-unsaturated (PUFA, up to 91.53%), followed by mono-unsaturated fatty acids (MUFA, up to 7.55%) and saturated fatty acids (SFA, up to 6.82%) [29]. Punicic acid is the most abundant fatty acid (ranging between 72.42 and 86.41%), followed by linoleic acid (4.11–11.32%) [32,33]. Of note, punicic acid is a conjugated α-linoleic acid that showed benefits against skin cancer [34], as well as anti-diabetic [35], anti-inflammatory, anti-obesity and antioxidant effects [36].

Oleic acid is the main MUFA (ranging between 3.63 and 7.12%), followed by palmitic acid (2.53–5.15%) and stearic acid (1.33–1.92%) [29,32,33].

In addition to fatty acids, PSO also counts sterols (in the range of 7.5 and 16.4 mg/g of oil), playing a critical role in the regulation of LDL cholesterol levels (Khemakhem et al., 2021). The main sterols found in PSO are β-sitosterol, ∆-5 avenasterol, campesterol, stigmasterol and sitosterol [29,32,37]. Seeds also contain triterpenes (0.99–3.13 mg/g of oil), such as cycloartenol and betulinol, and tocopherols (678.3–2627.4 μg/g of oil), for instance, α-, γ- and δ-tocopherols [32].

Regarding phenolic compounds, seeds are a source of anthocyanins, such as delphinidin, cyanidin and pelargonidin, and tannins, but less abundant compared to other parts of pomegranate [38].

Li et al. studied PSO composition through ultra-high-performance liquid chromatography coupled with quadrupole-Orbitrap high-resolution mass spectrometry; their results revealed that the major flavonoids were genistein, kaempferol, rutin, gallocatechin, luteolin, scutellarein and apigenin [39]. The total flavonoid content ranged between 2.5 and 7.5 mg rutin equivalent per g of extract [29].

Seeds also contain proanthocyanidins, responsible for their astringent and chelating metal ion activity [29], coumarins, fibers [31] and hydroxybenzoic acids [38]. Although few studies on seed oil amino acids content have been published, PSO seems to contain significant amount of glutamate, arginine, aspartate, phenylalanine and leucine [39,40].

### 2.3. Leaves

Pomegranate has glossy and narrow oblong leaves that are used to produce extracts or, in folk medicine, for infusion, tea or spices [41,42,43,44]. Pomegranate leaves aqueous/alcohol extract is rich in luteolin [17,45]. Of note, 23 compounds (11 phenolic acids, 8 tannins, 3 anthocyanins and 1 flavonoid) were identified by chromatographic technique, and the major compound resulted EA (43.14 ± 0.57 μg/mg of the fraction) [46]. Leaves are also a source of alkaloids and minerals in different percentage according to the age of the plant [47,48].

### 2.4. Flowers

*P. granatum* flowers are characterized by oval pink petals about 3 cm in diameter and bloom in summertime [49]. The flowers are a non-edible fraction, but their extract, by infusion or decoction, is traditionally used for the treatment of inflammation, diabetes, bacterial infection (including *Salmonella enterica*) and bronchitis [42,50,51].

From a phytochemical point of view, *P. granatum* flowers are rich in flavonoids (29.5 ± 0.8 mg quercetin equivalent/g dry weight), tannins (30.6 ± 0.6 mg catechin equivalent/g dry weight) and phenolic acids, such as gallic acid or EA (330.9 ±11.3 mg gallic acid equivalent/g dry weight) [50]. 

### 2.5. Peel

Pomegranate peel (PP) represents almost 50% *w*/*w* of the whole fruit and, although it is considered a by-product, it can be used to extract valuable bioactive compounds, so much so that PP is considered a “superfruit” waste product [7]. Major lipids available in pomegranate peel are linoleic acid, palmitic acid and oleic acid [17,45]. In addition to the primary metabolites, PP has elevated quantities of phytonutrients, such as flavonoids, ellagitannins and hydroxybenzoic acids, which are significantly higher than in the edible fraction [38,52,53].

Garcia and colleagues reported a phytochemical composition of a PP extract (PPE), obtained from pressurized liquid extraction. They documented the presence of 51 compounds, among which the major organic acids were citric acid, isocitric acid and dimethyl ester citric acid; moreover, they found significant levels of phenolic acids (i.e., chlorogenic acid, gallic acid, gallic acid derivate, ferulic acid, p-coumaric acid hexoside, phloroglucinol acid, cinnamoyl rhamnoside and homovanillic acid) and flavonoids, such as catechin, hyperoside, kaempferol, luteolin and luteolin derivates [54,55].

In regard to hydrolysable tannins, the chromatogram showed the presence of both gallotannins, such as galloyl-hexoside isomers, galloyl -HHDP-hexose and methyl gallate hexoside, and ellagitannins, such as punicalagin-α, punicalagin-β, punicalagin-γ, EA, valonic acid bilctone and brevifolin [8,54].

## 3. Pomegranate Extraction Techniques

### 3.1. Consolidated Extraction Methods

The most ancient and currently used pomegranate extraction method concerns PJ squeezing, usually performed by simply pressing the pomegranate fruit. However, the extreme richness in bioactive compounds of the entire pomegranate fruit other than PJ, including by-products of the PJ extraction process, stimulated the search for effective extraction techniques aimed at their exploitation. From a bioeconomy perspective based on feasibility and effectivity, by-products of PJ extraction, namely peel and seeds, attracted the greatest attention based on their concentration at relatively few manufacturing facilities, the impressive global amount, which was estimated in 2022 at the level of about 1.62 million tons [56], and the environmental burden caused by their disposal as waste residuals.

In a comprehensive review, Lampakis et al. reported on the consolidated extraction method, mainly applied to PP exploitation. Such a method foresees simple stirring and uses a few different types of solvents with varying degrees of selectivity, from water to methanol, ethanol and ethyl acetate. While affording smooth operation conditions, sufficient extraction yields of bound bioactive compounds, such as flavonoids, phenolic acids and proanthocyanidins, could be achieved only after long processes and correspondingly high levels of resource and energy consumption, which hindered the application of PP extraction beyond the experimental and laboratory scale [57].

### 3.2. Emerging Green Extraction Methods

In the comprehensive review cited in Section 3.1, Lampakis et al. also reviewed emerging extraction methods, such as pressurized liquid extraction, ultrasound-assisted and microwave-assisted extraction [57]. They found that the enhanced mass transfer rate due to solvent circulation and particle size reduction could accelerate the extraction rate of bioactive compounds and the antioxidant activity of the resulting extracts. However, none of the emerging methods have ever been documented at the industrial scale, while the standardization of operational parameters and proof of scale-up is still missing.

Another comprehensive review reported on emerging green extraction methods for both pomegranate seeds and peel, focusing on the potential optimization achievable after the application of innovative methods in support to conventional techniques, such as Soxhlet and stirring; however, they once again highlighted the lack of standardization and industrial applications [58]. Nevertheless, a simple, interesting innovation was represented by aqueous ball milling, as a PP extraction method showing the highest yield in punicalagins recovery. Such a method, extensively detailed in another article [59], used fresh PP, water as the only solvent (15 mL g^−1^ liquid:solid ratio) and worked at pH 7 and a temperature of 40 °C, affording fast (10–30 min) and efficient extraction of punicalagins. However, the authors recommended further research to explore the feasibility of the proposed method at the industrial scale.

Emerging green extraction methods of pomegranate by-products were further reviewed in 2022, focusing on the most studied one, i.e., ultrasound-assisted extraction—based on cavitation events in a liquid–solid mixture—which showed good effectivity at the laboratory scale but were hardly scalable; moreover, this was the same concerning microwave- and enzyme-assisted extraction [56]. The authors concluded that much research work remains to be performed, as the combination of different techniques, as well as the documented application scales, were laboratory-sized and the solid-to-liquid ratios were also generally quite low for the relatively consolidated ultrasound-assisted extraction method (generally 1:10 to more than 1:100). Interestingly, the main technologies used to encapsulate target compounds extracted from PP were extensively reviewed, including maltodextrins and pectin, as well as applications of PP extracts for the preservation and increase of shelf life of fresh and processed fruits, vegetables and beverages.

However, industry appeared to have advanced independentlyof fundamental research, offering food supplements on the market since the early 2010s, based on extracts from the whole pomegranate fruit, such as *Punicalagina granatum plus* from Antioxidantes del Mediterráneo S.L. (Spain), or *Keriba Forte* from Probelte Pharma (Murcia, Spain). Both supplements included Pomanox^®^, an ingredient in turn obtained according to a patented extraction method [60].

The latter patented method foresees, among other things, a mill/blender designed to extract pomegranate ellagitannins, specifically punicalagins, in weakly acidic water at moderate temperatures (lower than 30 °C) during 15 to 150 min, with solid to liquid ratios between 1:0.5 and 1:1 (however, PJ increases the liquid fraction by approximately 40% *w*/*w* of the mass of the entire fruit). The slightly acidic conditions were instrumental to avoid the hydrolyzation of punicalagins, which is favored by alkaline conditions. In this regard, it should be pointed out the decisive role of the pH level of the liquid–solid mixture used in PP extraction processes for the resulting amount of relative fractions of phenolic acids, hydrolysable tannins (such as punicalagins) and flavonoids, as well as of free and bound phenolics [61].

The above-cited method additionally included a fast heating step (70–100 °C during 1 to 120 s) for inactivate microorganisms and fungi-generated tannase enzyme, and adsorption of polyphenols and ellagitannins onto a resin and elution by weakly basic aqueous buffer, using sodium bicarbonate. A declared advantage of the method is the preferred extraction of punicalagins, which, contrary to ellagic acid, are readily water soluble. Moreover, spray-drying was indicated as the preferred method for the isolation of dry extracts, also associated with carriers suitable for the final use of the dry product, such as maltodextrins, soluble fiber, oat or barley beta-glucan rich soluble fiber, lactose, caseinates, etc.

### 3.3. Perspective: Hydrodynamic Cavitation

With the notable exception of the patented extraction method mentioned in Section 3.1, based on which the largely used nutraceutical ingredient Pomanox^®^ is currently developed, other methods, either consolidated or emerging ones, did not show sufficient effectiveness and efficiency, or even feasibility, beyond the laboratory scale. The tried and studied emerging methods include ultrasound cavitation-assisted extraction, pressurized liquid extraction, microwave-assisted extraction, enzyme-assisted extraction and aqueous ball milling. In this context, although it cannot be ruled out that any of the above-mentioned emerging extraction methods will be sufficiently upgraded up to the successful application at the relevant scale, based on the direct experience of the authors and the trends of the scientific literature, a specific technological roadmap is suggested to improve the exploitation of the valuable underutilized parts of the pomegranate fruit.

Hydrodynamic cavitation (HC) methods involve the creation of a periodic depression in liquid mixture, either by means of the active circulation of the liquid through a nozzle of suitable geometric shape, or moving mechanical parts, such as rotor–stator arrangements, in a still liquid. Vapor filled nano- and micro-bubbles form whenever the liquid pressure falls below the vapor pressure, and subsequently grow and implode under the external force produced by the recovered bulk liquid pressure. The implosion events release extraordinary intense energy pulses, and eventually, pressure shockwaves, hydraulic jets, extreme transient heating and chemical dissociation reactions [62].

HC-based technologies and related methods are emerging among the most effective, efficient and straightforwardly scalable in the field of the extraction of natural products, in comparison to both conventional methods and newer green technologies [63]. So much that a profound and long lasting impact on the whole natural product industry was recently predicted for these technologies [64], in addition to a key role in helping to achieve the sustainability development goals in a few different technical fields [65].

HC methods showed high process yields as single-unit operation systems applied to the extraction of natural products in water only at the pre-industrial scale [66], such as in the brewing field (extraction of cereals and hops), involving starch, proteins and polyphenols as the main constituents released into the water phase [67,68], conifer tree parts, involving polyphenols and volatiles [67], waste citrus peel involving pectin, polyphenols and volatiles [69], as well as with the extraction of fat, proteins and polyphenols from soybean [70] and whole almond seeds [71]. In all these applications to the extraction of natural products, HC processes afforded higher yields, even by tens of times, in comparison with competing methods, due also to the higher energy efficiency and the microbiological disinfection at temperatures much lower than conventional thermal processes [72,73,74].

Beyond the predictable comparatively higher extraction efficiency, the processing of pomegranate waste materials using HC methods, which has not been performed so far, is advisable based on the coexistence in PP of pectin and polyphenols. Pectin, largely available in the peel of fruits, such as citrus and pomegranate [58,75,76], revealed the ability, following HC-based extractions, to form stable complexes with flavonoids adsorbed onto its surface, whose porosity was greatly enhanced by cavitation processes, which were called IntegroPectin [69,77,78,79,80,81]. These chemically, morphologically and thermally stable complexes possess enhanced biological functions, including increased bioavailability and bioaccessibility, in comparison to pure molecules, as recently proven in vivo [82]. It is predictable that the same advantages would also occur with pomegranate, in terms of enhanced extraction rate, higher process yield, smaller particle size, increased solubility and bioavailability.

Further insight into the potential advantages of HC methods applied to the processing of pomegranate resources is provided in Section 5.

## 4. Beneficial Effects

**Figure 1:**
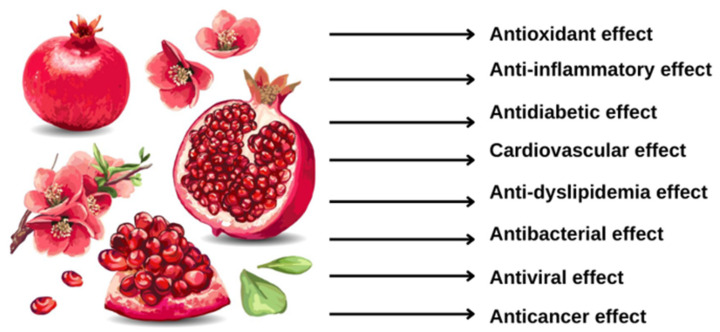
Pomegranates are rich in phytoconstituents which show numerous beneficial health proprieties, such as antioxidant, anti-inflammatory, antidiabetic and cardiovascular, along with anti-dyslipidemia, antibacterial, antiviral and anticancer activity.

Pomegranate exhibits beneficial health properties that have been investigated by clinical (Table 1) and preclinical (Table 2 and Table 3) studies, for the presence of numerous secondary metabolites (Figure 1).

### 4.1. Antioxidant Activity

It is well-known that pomegranate exhibits antioxidant properties, making it an interesting food for health and a valuable food additive. Indeed, food packaging industry uses PP powder to increase the shelf life of the foods, such as meat, bread and ice-cream [1,83,84].

In regard to the antioxidant profile, pomegranate reduces the reactive oxygen species and upregulates antioxidant enzymes levels, inhibiting nuclear factor-κB (NF-kB) and activating proliferator-activated receptor γ (PPAR-γ) [85,86]. In clinical trials, the consumption of pomegranate polyphenols reduced lipid peroxidation metabolites (such as malondialdehyde, MDA) and increased the total antioxidant capacity (TAC) and paraoxonase-1 (PON1) activity [87].

The administration of PJ to type 2 diabetic (T2D) patients significantly improved the antioxidant defenses. In particular, Sohrab et al. conducted a randomized, double-blind, placebo-controlled clinical trial on 44 T2D patients that was supplemented with 250 mL/day of PJ (corresponding to an amount of polyphenols equal to 345.87 µg catechin equivalent/mL). At the end of the study, blood sample analyses demonstrated that PJ ingestion promoted a significant increase of TAC and a significant decrease of MDA [88]. In another study, researchers confirmed that 200 mL/day of PJ (containing 2125 mg/L total polyphenols and 385 μg/mL flavonoids) for 6 weeks reduced the oxidative blood parameters, such as oxidized LDL, and increased the serum TAC and the PON1 in T2D individuals [87]. Finally, 50 g/day of a concentrated PJ, containing polyphenols in concentration of 6.3 mg/100 g, after 4 weeks allowed the increase of HDL levels and contributed to significantly enhance TAC, as well as reducing serum levels of IL-6 in 40 T2D patients [89].

### 4.2. Anti-Inflammatory Activity

Several studies demonstrated that pomegranate extracts elicited preventive effects against several kinds of inflammatory diseases [90,91,92]. In in vitro studies on LPS-activated RAW 264.7 macrophages, the treatment with a *P. granatum* flower extract (PGF) at concentrations of 10, 25, 50 and 100 μg/mL decreased inflammatory factors, such as NO, PGE_2_, IL-1β, IL-6 and TNF-α [92]. These effects were also evaluated by in vivo studies on rats, in which colitis was induced by treatment with 2,4-dinitrobenzene sulfonic acid (DNBS). PJ (400 mg/kg) and purified punicalagin (4 mg/kg), the amount of the latter corresponding to the content of punicalagin in PJ, showed preventive effects on the development of disease. In fact, at the end of the experimental protocol, the biochemical assays showed a reduction of neutrophils infiltration and nitric oxide levels, and an increment of superoxide dismutase (SOD) levels. Consistently, the pre-treatment prevented the histological damage provoked by DNBS and reduced IL-1β, IL-18, TNF-α and NF-κB gene expression. PJ and punicalagin ingestion, in particular, reduced NF-kB mRNA expression levels by about 84 and 64%, respectively [90]. The higher activity shown by PJ compared to purified punicalagin suggested that several phytochemical components of the juice contribute synergistically to the desired effect.

EA tested in mice suffering from ulcerative colitis (UC) and in a murine model of Crohn disease (CD) highlighted an interesting preventive profile. For example, balb/c mice were acutely treated with dextran sulfate sodium (DSS) in the amount of 5% of water daily intake and supplemented with EA (100 mg for day) for 7 days; in regard to the chronic model, C57BL/6 mice consumed a diet supplemented with EA (25 mg for day) during 4 cycles of DSS treatment (1% and 2% of water daily intake). The immunohistochemical, RT-PCR and Western blot results revealed that EA prevented, in acute as well as in chronic protocol, the evolution and the intensity of the disease and significantly reduced intestinal inflammation markers [93]. A reduction of COX-2, iNOS and other pro-inflammatory markers expression was observed in Wistar rats with trinitrobenzene sulfonic acid (TNBS)-induced colitis supplemented with EA (10–20 mg/kg for day). Moreover, an improvement of colonic health and a reduction of morphological alterations, provoked by TNBS, was also reported [94].

Of note, several in vitro studies showed that other secondary metabolites, such as urolithin A, punicalin, grantin B, strictinin and punicalagin could contribute to the anti-inflammatory activity [95,96]. Furthermore, punicic acid may participate to reduce the metabolic inflammation correlated to the obesity, through the activation of PPAR-γ [97].

Shukla et al. focused on the pomegranate extract administered to a murine rheumatoid arthritis model. The results clearly revealed that pomegranate extract reduced the inflammatory infiltration and markers (such as IL-6, IL-1β and TNF-α) in arthritic mice joints [98].

More interestingly, several clinical trials revealed the beneficial effects of pomegranate extracts against the inflammatory component associated to UC and rheumatoid arthritis. In this regard, 62 patients with UC were treated with a dose of 6 g/day of PP extract for 10 weeks and their symptoms were monitored using the Lichtiger Colitis Activity Index (LCAI). LCAI, recorded at baseline, after 4 and 10 weeks, suggested that the supplementation allowed a clinical improvement of UC symptoms, such as fecal incontinence, diarrhea and rectal bleeding [99]. 

In another study, 55 patients with rheumatoid arthritis were daily supplemented with 2 capsules of 250 mg pomegranate extract for 8 weeks and the progression of disease was monitored before and after the treatment using the Disease Activity Score 28 (DAS28) and Health Assessment Questionnaire (HAQ). The results indicated an amelioration of the disease, with a significant reduction of DAS28 points, due to a reduction of bloated joints count, pain intensity and blood ESR [100]. Further clinical trials revealed that pro-inflammatory markers, such as IL-6, high-sensitivity C-reactive protein (hs-CRP) and E-selectin, were also decreased by supplementation of PJ in T2D patients [89,101].

### 4.3. Anti-Diabetic Effects

Several works demonstrated that pomegranate is endowed with positive effects on glycemic homeostasis, and few authors reported that the supplementation with pomegranate extracts reduced blood glucose levels in different murine models of diabetes [102,103,104]. 

Huang et al. highlighted that the treatment of Zucker diabetic rats with PGF, containing high levels of gallic acid, in doses of 500 mg/kg for 6 weeks increased the PPAR-γ mRNA and GLUT4 mRNA expression, improving the insulin sensibility [104].

In addition, 250 and 500 mg/kg of PGF dose-dependently reduced the fasting blood glucose (FBG), together with an enhancement of lipidic profile and glutathione (GSH) levels in Wistar diabetic rats [105]. In regard to the possible mechanisms, it was hypothesized that the activation of insulin receptor (IRS) and glycogen synthase kinase 3 beta (GSK-3β) receptor, as well as the inhibition of the endoplasmic reticulum stress signaling pathway, was responsible for insulin resistance [106].

Based on the analysis of the PGF extract, other possible action mechanisms emerged, involved in the glycemic control. Tricetin, luteolin, punicalagin, EA, apigenin and granatin B showed a relevant inhibitory activity on α-glucosidase, α-amylase and lipase, similar to the antidiabetic drug Acarbose, thus reducing the absorption of glucose in the gastrointestinal tract [107].

Consistently with preclinical evidence, several clinical trials suggested that pomegranate extracts and the corresponding isolated bioactive compounds can contribute to reduced FBG in diabetic patients. In particular, 52 obese T2D patients, treated for 8 weeks with 3 capsules/day containing 1 g of PSO or with placebo, showed a significative reduction of FBG levels and increase of the GLUT-4 gene expression, without changes to insulin, HOMA-IR and HOMA-β levels [108]. Moreover, PJ administration reduced FBG levels in 85 T2D patients 3 h after the intake (1.5 mL/kg). It also reduced the insulin resistance and increased the β-cells function compared to a control group constituted of 50 healthy volunteers [109].

Likewise, 60 T2D patients involved in a placebo-controlled randomized blind trial received a supplementation with 5 g of pomegranate seed powder (PSP) twice a day for 8 weeks. Blood sample analysis revealed that FBG and glycated hemoglobin (HbA1c) levels were reduced [110]. In another study, 50 T2D patients treated with PJ in oral doses of 200 mL/day for 6 weeks showed lower levels of FBG, TC and LDL, along with an increase of PON1, an antioxidant enzyme that is downregulated in case of diabetes [111].

### 4.4. Cardiovascular Effects

The effects of different kinds of pomegranate extracts on cardiovascular system are undoubtedly the most explored. Vilahur et al. showed that the commercial pomegranate extract Pomanox^®^ (625 mg/day, titled in punicalagin 32.21%), reduced the coronary endothelial dysfunction in pigs fed with a high fat diet (HFD) [112]. A protective effect was also observed in Zucker diabetic fatty rats, after oral administration of a PGF extract for 6 weeks (500 mg/kg). The treatment reduced the triglycerides (TG) and fatty acids (FA) plasma levels and improved abnormal cardiac lipid metabolism [113].

The cardioprotective effects of pomegranate were demonstrated in even more drastic conditions. In a study, rats were supplemented with PJ (20 mL/day) during 29 days before receiving intraperitoneal injection of isoproterenol (85 mg/kg/day for 2 days). The exposure to PJ attenuated the tissue necrosis damage and diagnostic markers, such as creatine phosphokinase (CK) and lactate dehydrogenase (LDH) in blood samples. In the cardiac tissue, the supplementation also provoked an increment of reduced glutathione, vitamin C, SOD and catalase (CAT), suggesting a cardioprotective action [114].

In another study, the protective effect of pomegranate in rats exposed to a ton of cigarette smoke for 1 month was evaluated. The group was supplemented with PJ (containing 80 μM/day of polyphenols) for 1 week before the cigarette smoke exposure (1 h for 5 days/week) showed lower cardiac hypertrophy and aortic calcification compared to the control group, highlighting an anti-atherosclerotic action [115].

Clinical studies confirmed the above-described effects. In a randomized, placebo-controlled, double-blind clinical trial, which involved 45 adults with myocardial ischemia, the group supplemented with 240 mL/day of PJ for 3 months showed an improved myocardial perfusion up to 17%, reducing angina episodes by 50% in comparison with the control group, in which they increased by 38% [116].

In another study, 100 adults with a diagnosis of unstable angina or myocardial infarction were supplemented for 5 days with 220 mL/day of PJ, containing a polyphenol title of 840 mg/g dry weight and particularly rich in EA (88.8%). This integration confirmed the improvement of cardiac damage, the reduction of the incidence, length, gravity and score of angina episodes suffered by patients. Blood levels of TNFα, IL-6, MDA, troponin and hs-CRP were evaluated through biochemical assays and statistic results reported that PJ determined a significant reduction of serum troponin (79%) and MDA (29%) content in comparison with the control group [117].

PJ intake ameliorated even the histological condition of carotid artery stenosis, slowing down its progressive thickness in 15 patients who consumed 50 mL/day of PJ for 12 months, as well as in 289 patients supplemented with 240 mL/day of PJ for 18 months [118,119].

In addition to cardioprotective effects, pomegranate intake was also associated with a decrease of blood pressure; in particular, the intake of EA was associated with vasodilatation, due to the upregulation of eNOS and the mitigation of the reactive oxygen species. EA-related vasorelaxation was also studied in rat aorta [120], showing the involvement of an endothelium-dependent pathway [121].

Another putative mechanism involved in antihypertensive property is the inhibition of angiotensin converting enzyme (ACE). Of note, Wistar rats with streptozotocin-induced diabetes were pretreated with PJ (100 mg/kg/day and 300 mg/kg/day), 4 weeks before inducing hypertension with angiotensin II. Based on serum ACE activity evaluation and blood pressure measurements, the results revealed that PJ intake avoids the increment of blood pressure, probably reducing the oxidative stress and the ACE activity in comparison with non-treated animals [122]. In a clinical trial, it was also reported that in hypertensive patients, the ACE activity was reduced by about 36% following the treatment with PJ (50 mL/day, containing 1.5 mmol of total polyphenols) for 2 weeks [123].

In general, several clinical studies reported the lowering of systolic (SBP) and diastolic blood pressure (DBP) associated to a supplementation with PJs and PP extracts [124,125,126,127]. For example, 13 hypertensive men consumed 150 mL of PJ (presenting a total anthocyanins content of 5.8 mg/100 mL) after 12 h fasting; at baseline, and 4 and 6 h after PJ ingestion, their blood pressure level was measured and blood samples were collected. The results showed a significant blood pressure reduction associated to a reduction of serum E-selectin, CRP, IL-6, VCAM-1, and ICAM-1 [128].

### 4.5. Hypo-Lipidic Effects

A body of evidence demonstrates that pomegranate is endowed with beneficial effects in dyslipidemic disorders, positively correlated to obesity, metabolic syndrome and cardiovascular diseases.

Mice presenting a deficiency of Apolipoprotein E and LDL receptors (ApoE/LDLR^−/−^) and fed with a diet enriched in PSO reported a significant reduction of TC and TG plasma levels [129]. Michicotl-Meneses et al. focused on PJ effects in Wistar rats fed with HFD. The animals were treated with PJ (10 mL/kg for day) diluted into the water daily intake. The treatment showed that PJ, rich in phenolic compounds (6.17 mg gallic acid/mL) and in anthocyanins (0.191 mg cyanidine-3-glucoside eq/mL), increased HDL by 27% and lowered LDL by 39%, determining a marked reduction of cardiovascular risk (12–18%) [130].

In a clinical trial, PP extract was orally administered to 38 obese women in doses of 500 mg/day for 8 weeks and the results confirmed the significant reduction of TC, LDL, TG and CRP [131]. Likewise, Anoosh et al. divided 36 patients into 3 groups: 1 group was treated with *tabrizy* variety pomegranate, 1 group with *black* variety and 1 group with lovastatin, as control group. PJ-supplemented groups showed reduced LDL levels, in particular in the first group it decreased from 158.25 to 130.50 mg/dL, the second group from 150.50 to 130.92 mg/dL and in the control group from 138.33 to 111.58 mg/dL [132].

It is well-recognized that lipid imbalances could provoke atherosclerosis, characterized by alteration of blood vessel and accumulation of lipidic molecules and inflammatory cells (the so-called “foam cells”). Several works focused on pomegranate role in attenuating atherosclerosis progression due to its antioxidant activity, which avoids macrophages and lipoprotein oxidation and lipid deposition [133].

Salama et al. conducted a study on the antiatherogenic proprieties of pomegranate in a rodent model. In particular, 28 albino rats were divided into groups and fed with HFD or HFD + PP powder supplementation. HFD group showed elevated inflammatory markers and lipidic profile associated to metabolic disorder, weight gain and alterations in vascular structures. The nutraceutical supplementation reduced TC and IL-6 levels and showed a protective action against atherosclerosis and cardiovascular risk, reducing the aorta damage induced by HFD [134].

Manickam et al. worked on a placebo-control trial on ApoE^−/−^ mice treated with hydro-ethanolic extract of PP. Animals were fed with a high cholesterol diet and orally treated with 200 mg/kg PP or placebo by gavage for 12 weeks; blood samples and aorta tissue were collected and used for analysis in vitro. Together with the reduction of glucose, inflammatory markers, TC and TG, the treatment also stabilized the aorta necrosis area, facilitating the plaque remodeling and increasing the collagen content [135].

Aviram et al. carried out studies both on human adults and on rodents. In preclinical study, apolipoprotein E-deficient mice received 0, 6.25, and 12.5 mL of PJ (containing 0, 0.175 and 0.350 mmol of total polyphenols) in their water daily intake for 11 weeks. The authors highlighted that the treatment reduced LDL oxidation up to 90% and the atherosclerosis vessel damage by 44%. In the clinical trial, human volunteers were supplemented with 50 mL/day and another group with 20–80 mL/day. Blood sample analyses indicated that the PJ ingestion reduced LDL aggregation and increased PON1 activity, which acts against peroxidation [136].

Several works demonstrated that the treatment with PGF reduced the amount of lipid droplets and adipose tissue, whereas punicic acid elevated the PPAR-α and PPAR-γ expression in 3T3-L1 adipocytes, improving glucose homeostasis and lipidic metabolism, thus suggesting a beneficial effect on this district with interesting consequences on the metabolic profile [97,137].

### 4.6. Neuroprotective Effects

A growing body of knowledge associated EA, derived from the ellagitannin punicalagin, with highly promising actions in the prevention and treatment of neurodegenerative diseases; the mechanisms underlying these activities included not only direct antioxidant and anti-inflammatory activities, but also the regulation of the metabolism of neurotransmitters.

Recently, an in vitro approach highlighted an interesting cholinesterase inhibitory activity of PP methanol and ethanol extracts, observing that this biological effect was positively associated with their bioactive metabolite content [138].

In vivo experiments were also performed. Amri et al. investigated the beneficial effects of pomegranate extracts obtained with several parts of the plant (seeds oil, leaves, juice and peel) in high fat–high fructose diet-induced-obese rats, in terms of brain cholinesterase activity and brain oxidative stress. The results confirmed the neuroprotective effects of pomegranate extracts obtained by means of the inhibition of cholinesterase and the increase of antioxidant capacity [139]. Consistently, EA, extracted from PP, also protected hippocampal CA1 pyramidal neurons in a rat model of Alzheimer’s disease [140]. Likewise, in rats with Parkinson’s disease, EA showed reduced activity of MAO-B and an increase in Nrf2 [141].

Finally, the oral administration of EA delayed the onset and reduced the progression of the disease, in an experimental autoimmune encephalomyelitis, the most common model for multiple sclerosis characterized by inflammatory cell infiltration into the central nervous system and demyelination [142].

### 4.7. Antibacterial Activity

*P. granatum* is correlated with antifungal and antibacterial functions due to the presence of punicalagins, which inhibit the growth of Gram+ and Gram− bacterial species, such as *Candida albic*, *Pseudomonas aeruginosa*, *Escherichia coli* or *Staphylococcus aureus*.

Gosset-Erard et al. investigated the antimicrobial activity of PP ethanol extract by the agar diffusion assay against *Pseudomonas aeruginosa* (ATCC 9027 strain) and *Staphylococcus epidermidis* (ATCC 12228 strain) [143]. After incubation, they observed an inhibition zone of 20 mm that confirmed the *P. granatum* activity and, analyzing the extract through UHPLC, they identified punicalagin α and β as the main responsible for such activity [143,144].

*P. granatum* alcohol and aqueous leaves extract exhibited anti-fungal properties against the common fungi *Candida albicans*, *Aspergillus niger* and *Penicillium chrysogenum*. The extract, rich in maltol as the main phytoconstituent, showed also an anti-dandruff activity and promoted hair growth [43].

The antimicrobic action of *P. granatum* extracts was also exploited to produce mouthwash. Double-blind clinical studies tested pomegranate-based mouthwash in order to reduce plaque of *Streptococcus mutans* in the oral cavity. Adults or students used pomegranate mouthwash or gel in comparison with a chlorhexidine-based product and the results were encouraging [145,146,147].

### 4.8. Antiviral Activity

SARS-CoV-2 infection produces a respiratory disease (Coronavirus 2019 disease) and proceeds through the virus attachment due to the presence of viral proteins, for instance, Mpro protease, 3CL protease and Spike protein, allowing the access into human cells and the virus replication. Spike protein, in particular, is one of the main ones responsible for the virus infection because of its interactions with receptor binding domain (RBD) and ACE2 receptors, abundantly expressed in heart, brain, kidneys and respiratory system. Possible targets are the inhibition of protease activity, reducing the virus replication, or the inhibition of SARS-CoV-2-Spike interactions with ACE2 or RBD receptors. In recent years, several studies demonstrated that certain polyphenols can reduce the replication and diffusion of SARS-CoV-2, including studies on the interaction of pomegranate extracts with ACE receptors [148,149].

Tito et al. focused on the inhibition of SARS-CoV-2 interaction with ACE2 receptors induced by PP extract, using a cellular model with Human kidney 2 cell (HK2), presenting a remarkable amount of ACE2 receptors. The authors found that PP extract inhibited the Spike-ACE2 interaction by 74% at the concentration of 0.04 mg/mL and 1 mg/mL, as well as diminished the ACE2 and TMPRSS2 gene expression, thereby reducing the virus access [150]. Moreover, molecular docking analyses allowed the investigation of the interaction between the Mpro protease and the main pomegranate polyphenols, showing that punicalagin and EA were the most effective polyphenols [148]. 

Suručić et al. also analyzed the antiviral activity of PP extract and its major polyphenols using in silico and in vitro models. Molecular docking studies revealed that punicalin, followed by punicalagin, EA and urolithin A created the best interaction with S-protein, thus inhibiting the virus infectivity. PP extract and its main polyphenols (punicalin, punicalagin, EA, urolithin A and gallic acid) were also tested by in vitro model using an ELISA kit which evaluated the binding between RBD and ACE2 receptor of host cell. The 50% inhibition concentration (IC50) was tested in the range of concentrations of 62.5–1000 μg/mL, with the results indicating that the PP extract showed a dose-dependent and more effective inhibition of Spike-ACE2 interaction than its single polyphenols (83.25%), leading to hypothesize a possible synergistic activity by pomegranate polyphenols. Punicalin was shown as the most effective among polyphenols (78%), confirming the docking results [151].

Finally, a randomized blind placebo-control clinical study on 182 volunteers with COVID-19 highlighted that a treatment with PJ (200 mL, 3 times a day) reduced symptoms such as fever, cough, diarrhea, taste and smell alteration, abdominal pain and nausea [152].

### 4.9. Anticancer Effects

Pomegranate inhibits the growth of several types of cancer cells, including prostatic, cervical, breast, pancreatic, lung, colonic and hepatocellular cell lines, through different mechanisms [153,154].

Pomegranate breast anticancer properties were attributed to its antiproliferative effect correlated to anti-aromatase and anti-estrogenic activity. Indeed, it is well-known that high levels of estrogens are one of the main risk factors for the growth of breast tumor [155]. Estrogen release is modulated by two nuclear receptors, ER-α and ER-β, that represent two of the main anticancer targets, for instance of the chemotherapeutic drug tamoxifen. Considering that ER-α promotes the breast cancer proliferation and ER-β blocks the progression, the ER-α antagonism is one of the most effective anticancer strategies [20]. Other possible targets are represented by aromatase and 17β-hydroxysteroid dehydrogenase enzymes, involved in estrogens biosynthesis [27].

In recent years, *P. granatum* polyphenols were tested for their anti-proliferative effects. Kim et al. discovered that polyphenols from PSO, fermented PJ and PP aqueous extract inhibited dehydrogenase activity and the growth of ER + MCF-7 and ER-MDA MB231 breast cancer cell lines [27]. In vitro studies demonstrated that among pomegranate tannins, the most potent enzyme-inhibitor is urolithin B, followed by gallagic acid [156].

Ozkan et al. tested eight different *turkey* pomegranate varieties on MCF-7 breast cancer cell lines and MCF-10A breast fibrocystic cell lines. Cells were treated with PJ in different concentrations (750, 250, 100, 50, 5, 2.5, 1 μg/mL) and assays were performed to control the cytotoxic and vitality effect. All the *P. granatum* varieties showed an antiproliferative effect on cancer cells, reducing viability without manifesting toxic effects. The most effective PJ was extracted from the *izmir* variety (IC_50_ 49.08 µg/mL), which contained high amounts of anthocyanins (69.76 ± 8.02 μg cyanidin chloride/g extract) and punicalagin (992.09 ± 174.53 μg/g extract) [157].

Likewise, Jeune and colleagues studied the anticancer activity of pomegranate extract, combined with the isoflavone genistein, in breast cancer cells MCF-7. Genistein is a natural constituent in soybean and possesses binding capacity to ER receptor. After 24 h treatment with 5 different PJs and genistein in single and combination treatments, the cytotoxic effect and the growth reduction were quantified. Results revealed that pomegranate extracts *plus* genistein were able to induce cell apoptosis [158]. 

Consistently, punicalagin, EA and total pomegranate tannins showed apoptotic and antiproliferative actions on human colon, oral and prostate cancer cells [159]. Dahlawi et al. demonstrated that PJ extract induced apoptosis in leukemia cell lines, testing PJ extract on eight different leukemia cell lines and a control cell line [160], while Li et al. evaluated that the pomegranate leaves extract induced apoptosis in lung cancer cells, along with the concentration-dependent arrest of the cell cycle in G2/M phase [44].

Antioxidant and anti-inflammatory effects contributed to the anticancer action. Several studies found that the overexpression of COX2, strictly associated to cancer metastasis, could be significantly attenuated by the treatment with pomegranate extract, which was rich in polyphenols (107.5 ± 3 mg/g of tannic acid equivalents) [160,161,162]. The pomegranate-induced reduction of pro-inflammatory mediators was attributed a fundamental role in the contrast to colon or prostate cancer cell metastasis [163,164].

It is well-known that the NF-kB pathway is a fundamental actor in the oncogenesis processes and cell proliferation [20,165]. In the case of prostate cancer, preclinical analysis demonstrated that pomegranate inhibited the growth of tumor cells, by cell cycle arrest and induction of apoptosis [166,167]. Moreover, ellagitannins were found in high amounts in prostate tissue, suggesting that pomegranate metabolites could play an anticancer role in this tissue. Of note, González-Sarrías et al. focused on urolithins accumulation in prostate gland following PJ ingestion. A total of 63 volunteers with prostate cancer consumed 200 mL PJ/day for 3 days before surgery. The large presence of metabolites such as urolithin A, urolithin B and dimethyl EA pointed out their role in the beneficial effect against prostate cancer [168]. 

According to preclinical studies, in men with prostate cancer, the administration of 8 ounces of pomegranate juice by mouth daily (Wonderful variety, equivalent to 570 mg total polyphenol gallic acid equivalents daily) slowed down the blood increment of prostate-specific antigen (PSA), a well-known marker of cancer progression [169,170].

Furthermore, a study on patients, who were treated with 2 capsules/day of 300 mg of pomegranate extract (containing 40% polyphenols and 27% punicalagin) for 6–7 weeks, demonstrated the protective pomegranate effect in preventing radiotherapy damage at the skin level [171]. Indeed, it is known that the most frequent side effect of radiotherapy is represented by mucositis and dermatitis.

In general, pomegranate showed remarkable protective effects from ultraviolet (UV) irradiation, preventing ROS formation and inflammation, edema, hyperplasia, immunosuppression, photoaging and skin cancer [172]. The consumption of a pomegranate fruit extract standardized to punicalagins in doses of 5 to 60 mg/L was shown to protect human skin fibroblasts from cell death after UV exposure in a dose-dependent manner, due to a diminished activation of the pro-inflammatory transcription factor NF-kB, a downregulation of proapoptotic caspase-3 and an increased G0/G1 phase, associated with DNA repair, suggesting the use of pomegranate extracts in topical applications [172].

**Table 1:** Clinical studies.

Activity	Plant Part	Dosage	Design of study	Outcomes	Ref.
Antioxidant	PJ	50 g/day for 4 weeks.	Placebo-controlled RCT on 40 T2D	↓ IL-6, ↑ TAC	[89]
	PJ	200 mL/day for 6 weeks	Placebo-controlled RCT on diabetic patients	↓ lipid oxidation, ↑ TAC	[87]
	PJ	250 mL/day for 12 weeks	Placebo-controlled RCT on 44 patients with T2D	↑ TAC, ↓ MDA	[88]
Anti-inflammatory	PPE	6 g PPE/day for 10 weeks	Placebo-controlled RCT on 62 adults with UC	↓ UC symptoms (i.e., fecal incontinence)	[99]
	PE	2 capsules of 250 mg PE/day for 8 weeks	Placebo-controlled RCT on 55 rheumatoid arthritis patients	↓ joints pain, ↓ inflammatory markers	[100]
	PJ	250 mL/day PJ	Placebo-controlled RCT on 50 patients with T2D	↓ hs-CRP and IL-6	[101]
Antidiabetic	PSO	3 g/day per 8 weeks	Placebo-controlled RCT on 52 obese T2D patients	↓ FBG level, ↑ GLUT-4 expression	[108]
	PJ	1.5 mL/kg	Placebo-controlled RCT on 85 T2D patients	↓ FGB levels and insuline resistance, ↑ β-cell function	[109]
	PSP	5 g twice a day for 8 weeks	Placebo-controlled RCT on 60 T2D patients	↓ FBG and Hemoglobin A1C	[110]
	PJ	200 mL/day for 6 weeks	Placebo-controlled RCT on 50 T2D patients.	↓ FBG, cholesterol and LDL	[111]
Cardiovascular	PJ	240 mL/day for 3 months	Patients with coronary heart disease	↑ myocardial perfusion (17%) ↓ frequency of angina episodes (50%)	[116]
	PJ	220 mL/day for 5 days	Patients with heart diseases	↓ frequency, duration, severity of angina	[117]
	PJ	50 mL/day for 12 months	15 health patients	↓ systolic blood pressure and the lipids peroxidation	[118]
	PJ	240 mL/day for 18 months	289 patients with coronary heart diseases	↓ carotid thickness progression	[119]
	PJ	50 mL/day for 2 weeks	Hypertensive adults	↓ ACE activity (36%)	[123]
	PPE	250 mg/twice in a day for 8 weeks	Placebo-controlled RCT on T2D patients	↓ systolic and diastolic blood pressure, ↑ lipidic profile	[124]
	PJ	200 mL/day for 6 weeks	Placebo-controlled RCT on 60 health adults	↓ blood pressure, ↑ lipidic parameters	[125]
	PJ	330 mL/day for 4 weeks	Placebo-controlled RCT on 51 adults	↓ blood pressure	[126]
	PJ	150 mL/day for 2 weeks	Placebo-controlled RCT on 21 adults	↓ systolic and diastolic pressure, — inflammatory and lipidic profile	[127]
	PJ	150 mL/day	Placebo-controlled RCT on 13 men	↓ systolic and diastolic pressure, — IL-6, E-selectin, ICAM-1, CRP levels	[128]
Hypo-lipidic	PPE	500 mg for 8 weeks	Placebo-controlled RCT on obese female volunteers	↓ cholesterol, triglycerides, LDL, body weight and blood pressure	[131]
	PJ	200 mL/day for 4 weeks	Placebo-controlled RCT on 24 subjects treated with PJ or lovastatin	↓ cholesterol and LDL	[132]
	PE	1 g/day PE + 20 mg/day simvastatin	Placebo-controlled RCT on volunteers with hypercholesterolemia	↓ triglycerides, cholesterol and ROS in blood samples, ↑ protection against atherosclerosis	[133]
	PJ	50 mL/day or 20–80 mL/day	Healthy adults	↑ TAC, ↓ LDL oxidation and aggregation	[136]
Antibacterial and antifunginal	HAP	15 mL/day	Placebo-controlled RCT on 60 healthy adults	↓ dental plaque	[147]
Antiviral	PJ	200 mL/3 times a day	182 adults presenting SARS-CoV-2 infection	↓ COVID-19 symptoms	[152]
Anticancer	PJ	200 mL/day for 3 days	63 patients with prostate cancer or benign prostate hyperplasia	— CDKN1A, M Ki-67, c-Myc mRNA expression	[168]
	PJ	70 mg total polyphenol/8 once for day	Men with prostate cancer	↑ PSA doubling time from 15 months to 54 months	[170]
	PE	300 mg/2 time a day	Patients treated to prevent radiotherapy-induced mucositis and dermatitis	↑ defense from radiotherapy damages	[171]

*Abbreviations:* ↓ Reduction; ↑ Increment, — no change; IL-6: Interleukin-6; TAC: Total Antioxidant Capacity; MDA: Malondialdehyde; UC: Ulcerative Colitis; hs-CRP: high-sensitivity C-Reactive Protein; FBG: Fasting Blood Glucose; GLUT-4: Glucose transporter type 4; LDL: Low Density Lipoproteins; ACE: Angiotensin Converting Enzyme; ICAM-1: Intracellular Cell Adhesion Molecule; ROS: Reactive Oxygen Species; RCT: randomized controlled trial; T2D: type 2 diabetic; PJ: Pomegranate juice; PPE: Pomegranate peel extract: PE: Pomegranate extract; PSO: Pomegranate seed oil; PSP: Pomegranate seed powder; HAP: hydroalcoholic extract.

**Table 2:** In vitro preclinical studies.

Activity	Plant Part	Dosage	Design of Study	Outcomes	Ref.
Anti-inflammatory	PGF	10–100 μg/mL	LPS-induced RAW 264.7 macrophages	↓ inflammatory markers (i.e., NO, PGE2_,_ IL-1β, IL-6 and TNF-α)	[92]
Antidiabetic	Purified compounds from PGF		α-glucosidase, α-amylase and lipase assays	↓ α-glucosidase activity	[107]
Antibacterial and antifunginal	PPE	0.3–1.20 µg/mL	*P. aeruginosa* (ATCC 9027) and *S. epidermidis* (ATCC 12228)	↓ bacterial growth	[143]
	Leaves alcohol extracts	3–10% extract in distilled water.	*C. albicans*, *A. niger* and *P. notatum*	Anti-fungal and antidandruff activities	[43]
Antiviral	PPE	0.04 mg/mL	Human kidney 2 cell (HK2)	↓ Spike-ACE2 interaction and ACE2 and TMPRSS2 gene expression	[150]
	Polyphenols	-	Molecular docking studies	Punicalagin and EA were the most effective on Mpro interaction	[148]
	PPE and purified polyphenols	62.5–1000 μg/mL	Molecular docking studies and ELISA kit	↓ interaction between S protein and ACE2 receptor	[151]
Anticancer	PJ	-	Leukemia cell lines	↓ Cell proliferation	[160]
	Pomegranate leaves extract	0–200 μg/mL for 24, 48 and 72 h	Lung cancer cell lines (A549, H1299), mouse Lewis lung carcinoma (LL/2)	Arrest of cell cycle progression in G2/M phase	[44]
	PE	5–60 mg/L	UVA- and UVB-damaged human skin fibroblast cells (SKU-1064)	↓ pro-inflammatory transcription factor NF-kB, ↓ caspase-3, ↑ G0/G1 phase associated with DNA repair	[172]

*Abbreviations: *↓ Reduction; ↑ Increment, — no change; LPS: Lipopolysaccharides; NO: nitric oxide; PGE2: prostaglandin E2; IL-1β: interleukin-1β; IL-6: Interleukin-6; TNF-α: tumor necrosis factor-α; ACE2: Angiotensin Converting Enzyme 2; NF-kB: nuclear factor-kB; PGF: Pomegranate flowers extract; PPE: Pomegranate peel extract; PJ: Pomegranate juice; PE: Pomegranate extract; EA: Ellagic Acid.

**Table 3:** In vivo preclinical studies.

Activity	Plant Part	Dosage	Design of Study	Outcomes	Ref.
Anti-inflammatory	PJ and purified punicalagin	400 mg/kg (PJ) 4 mg/kg (purfied punicalagin) for 18 days	Sprague-Dawley rats affected by colitis using DNBS	↓ DNBS damage, inflammatory genes (i.e., TNF-α, IL-1β, IL-18 and NF-κβ)	[90]
	PE	13.6 mg/kg and 34 mg/kg for 10 days	Mice with rheumatoid arthritis	↓ IL-6, IL-1β and TNF-α levels, ↑ joint infiltration	[98]
	EA	100 mg/day for 7 days in acute model, 25 mg/day for 56 days in chronic model	Female mice with UC induced by DSS	Prevention, in acute as well as in chronic protocol, of the progression of the UC, ↓ inflammatory intestinal markers (IL-6, COX-2, iNOS and TNF-α)	[93]
	EA	10–20 mg/kg for 48 h	Wistar rats TNBS-induced colitis	↓ expression of COX-2, iNOS and other pro-inflammatory markers and morphological alterations	[94]
	PE and urolithin-A	250 mg/kg (PE) or 15 mg/kg (urolithin-A) for 25 days	Fisher rats with DSS-induced intestinal damage	↓ Inflammatory markers expression	[95]
	PSO	1 g/100 g diet for 1 month	Male prediabetic mice	↑ expression of PPAR-γ, ↓ FBG	[97]
Antidiabetic	Pomegranate seed extract	150–600 mg/kg	STZ-induced diabetic rats	↓ FBG	[102]
	PSO	1 g/kg/day for 12 weeks	C57Bl/J6 HFD mice	↓ body weight gain, ↑ insulin sensitivity	[103]
	PGF	500 mg/kg/day for 6 weeks	Zucker diabetic fatty rats	↓ FBG, ↑ PPAR-γ and GLUT4 mRNA expression	[104]
	PGF	250 mg/kg and 500 mg/kg for 21 days	Diabetic Wistar rats	↓ FBG, cholesterol, ↑ triglycerides, GSH, LDL, VLDL and tissue LPO levels, — HDL-C and antioxidant enzymes	[105]
	PGF	50–100 mg/kg for 4 weeks	STZ-induced diabetic rats	↓ weight gain and FBG, ↑ insulin sensitivity	[106]
Cardiovascular	PE (Pomanox^®^)	625 mg/day (200 mg punicalagin/day)	HFD-fed pigs	↓ vascular damage, ↑ oxidative stress	[112]
	PGF	500 mg/kg for 6 weeks	Zucker diabetic fatty rats	↓ lipid absorption and fatty acids	[113]
	PJ	20 mL/day for 30 days	Rats (IP)-induced cardiac damage	Protection of the hearts, ↓ oxidative stres	[114]
	PJ	80 μM/day of polyphenols for 1 month	48 rats exposed to cigarette smoke for one month.	Protection from cigarette damages, as aortic calcification, or cardiac hypertrophy	[115]
	PJ	100–300 mg/kg for 4 weeks	Diabetic Wistar rats with AngII-induced hypertension	↓ blood pressure	[122]
Hypo-lipidic	PSO	Diet enriched	ApoE/LDLR^−/−^ mice	↓ LDL and TG plasma level, — atherosclerosis progression	[129]
	PJ	10 mL/kg for 8 weeks	HFD-fed Wistar rats	↓ blood pressure, LDL and pro-inflammatory cytokines, ↑ HDL levels, E-selectine and adiponectin	[130]
	Pomegranate peel powder	0.5 g/kg for 4 weeks	28 HFD-fed albino rats.	↓ inflammatory markers, cholesterol and LDL, ↓ aorta alteration and cardioprotective effect.	[134]
	PPE	200 mg/kg for 12 weeks	ApoE^−/−^ mice	Stabilization of the aorta necrosis area, facilitation of the plaque remodeling and the collagen content, necrosis area	[135]
	PJ	0–12.5 mL for 11 weeks	*Apolipoprotein E-deficient* mice	↓ LDL oxidation (90%) and atherosclerosis vessel damage (44%)	[136]
Neuroprotective	PSO, PL, PP, PJ	PSO: 2 mL/kg/day; PJ, PP and PL: 250 mg/kg/day	Standard diet or HFD diet rats	↓ cholinesterase activity, ↑ antioxidant capacity	[139]
	EA	50 mg/kg for 30 days	STZ-induced sporadic Alzheimer’s desease rats	↑ cognitive behavior, protection in hippocampal CA1 pyramidal neurons, ↓ inflammation and oxidative markers	[140]
	EA	50 mg/kg/day for 1 week	Intrastriatal 6-OHDA-lesioned rats	↓ MDA, ROS, DNA fragmentation and MAO-B activity	[141]
	EA	10 mg/kg /day for 12 or 18 days	Lewis rats with autoimmune encephalomyelitis	↓ progression of the disease	[142]

↓ Reduction; ↑ Increment; — no change. *Abbreviations:* DNBS: 2,4-dinitrobenzene sulfonic acid; TNF-α: tumor necrosis factor-α; IL-1β: interleukin-1β; IL-6: Interleukin-18; IL-6: Interleukin-6; Nf-kB: nuclear factor-kB; COX-2: Cyclooxygenase-2; iNOS: Inducible nitric oxide synthase; PPAR-γ: proliferator-activated receptor γ; FBG: Fasting Blood Glucose; GLUT-4: Glucose transporter type 4; GSH: glutathione; LDL: Low Density Lipoproteins; VLDL: Very Low Density Lipoproteins; HDL: High Density Lipoproteins; TG: triglycerides; MDA: Malondialdehyde; ROS: Reactive Oxygen Species; MAO-B: Monoamine oxidase B; UC: Ulcerative colitis; DSS: dextran sulfate sodium; TNBS: trinitrobenzene sulfonic acid; STZ: streptozotocin; HFD: High Fat Diet; IP: isoproterenol; PGF: Pomegranate flowers extract; PPE: Pomegranate peel extract; PJ: Pomegranate juice; PE: Pomegranate extract; EA: Ellagic Acid; PSO: Pomegranate seed oil; PL: Pomegranate Leaves; PP: Pomegranate peel.

## 5. Pharmacokinetics of Ellagitannins

About 100 ellagitannins are available in the pomegranate fruit and plant, the most abundant of which are punicalagins, punicalin A and punicalin B, which are also responsible for most of the antioxidant activity in vitro [4,14].

After the intake of PJ or other extracts, the main ellagitannins, punicalagins, are almost undetectable, whereas EA and its metabolites are detected in the blood. Indeed, it was suggested that ellagitannins are hydrolyzed by ellagitannase (ellagitannin acyl hydrolase) to release EA [173]. However, Cerda et al. found small amounts of punicalagin in the plasma of rats treated with standard diet containing 6% of punicalagin. Its plasma concentration reached the maximum value after 8 days, then remained constant during the supplementation; the authors also detected its metabolites, such as EA and glucuronide derivatives [174]. In a study on the bioavailability of ellagic acid in human plasma, it was shown that after the intake of 180 mL PJ (containing 25 mg of EA and 318 mg of punicalagins), 31.9 ng/mL of EA was found after 1 h in the human blood, but was rapidly eliminated by 4 h [175]. Similar results were published by Lei and colleagues, who reported a maximum EA plasma concentration of 33.8 ng/mL less than 1 h after the administration of 800 mg of pomegranate extract (containing 21.6 mg of EA and 330.4 mg punicalagins). Again, EA was not detectable 4 h after the oral administration [176].

Regarding the preclinical studies, Doyle and colleagues, for the first time, observed the absence of EA in feces or urine of rats, but small amounts were detected in the feces of germ-free animals; conversely, they detected two metabolites (3,8-dihydroxy-6H-dibenzo[b,d]pyran-6-one, re-called Urolithin A and one not identified), suggesting that both metabolites were of microbiota origin [177]. Other authors reported that, in mice, EA was excreted in urine and no microbiota metabolites were detected in the blood [178]. More recently, an oral administration of 50 mg/kg of EA showed a plasma peak after about 30 min and a maximum concentration of 93.6 ng/mL [179]. Of note, EA is poorly absorbed, its bioavailability being about 4%. In summary, after EA ingestion, a small amount of free compound can be absorbed in the stomach, while the rest is absorbed in the intestine. Conversely, ellagitannins are resistant to gastric metabolism and their hydrolysis occurs in the intestine, giving free EA that can be absorbed through a passive diffusion process, driven by the concentration gradient.

**Figure 2:**
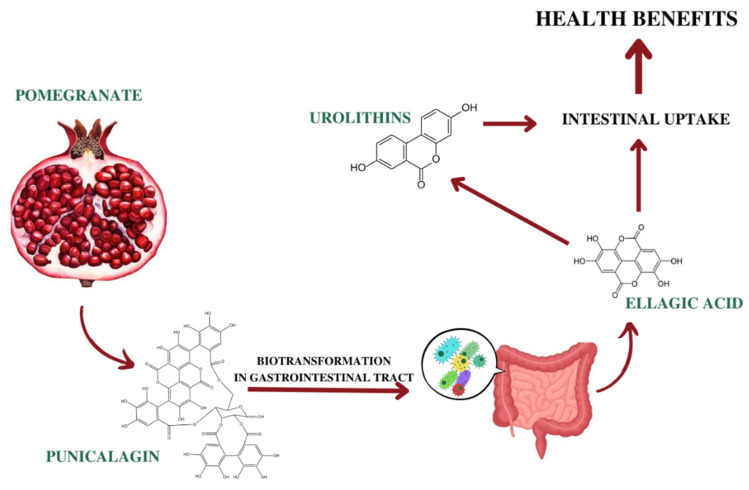
Ellagitannins, such as punicalagin, are hydrolyzed in the small intestine, giving free EA, that can be absorbed through a passive diffusion process. Unabsorbed EA could be converted by microbiota in the colon in urolithins [181].

Interestingly, Gonzalez-Sarrias and colleagues described a saturation process in the small intestine, when high doses of ellagitannins were used. In fact, they observed that the increase of the EA dose did not enhance the plasmatic concentration, compared to lower dose [180]. Finally, unabsorbed EA could be converted by microbiota in the colon in urolithins, which were identified as the ultimate conveyors of most of beneficial biological effects in humans [181] (Figure 2).

Taken together, clinical and preclinical evidence points out a poor water solubility and a limited absorption rate of EA, which greatly compromise its oral bioavailability and the clinical use of pomegranate extracts. Therefore, research and the pharmaceutical industry are committed to developing several strategies to address the many drawbacks associated with EA in vivo absorption, to enhance the biopharmaceutical properties. Here, we presented the most investigated solutions studied in the last years.

Innovative nanoparticle-based approaches by using microspheres, nanoparticles, pH-dependent microassemblies or nanogels have been described. In particular, the green synthesis of nanoparticles based on certain metals, such as gold, silver, zinc, and copper, mediated by PPE, with polyphenols acting as reducing agents, received intense attention in recent times, as such nanoparticles showed a far greater in vitro bioavailability of the bioactive compounds available in PPE, both punicalagin and EA, along with the possibility to carry specific drugs to target tissues [182]. In the same study, it was pointed out that few products based on such nanoparticles are already available on the market, while several in vivo and clinical trials are underway.

Moreover, the water solubility and dissolution rate can be improved through a fast cooling and precipitation in the presence of polymeric matrices, which allows for the obtaining of an amorphous solid dispersion. This is one of the most promising techniques. Cellulose derivatives, such as carboxymethylcellulose acetate butyrate (CMCAB), cellulose adipate propionate and hydroxypropyl methylcellulose acetate succinate (HPMCAS), are the most common matrices employed to obtain amorphous solid dispersions with EA. Dissolution tests demonstrate a rapid and almost complete release of EA at pH 6.8 from HPMCAS as well as CMCAB. However, amorphous forms tend to recrystallize; indeed, the instability is the major drawback and can lose the advantage of these forms [183].

Complexes with cyclodextrins represent another suitable strategy to improve EA bioavailability. Specifically, cyclodextrins are cyclic oligosaccharides formed by α-glucopyranose units (from 6 to 12), which have a hydrophobic central cavity and are hydrophilic on the outside, allow for producing inclusion complexes with a wide range of solid, liquid or gaseous substances and improve their water solubility. Dissolution tests suggested that EA is released from cyclodextrins complexes faster and more completely than free EA. Chudasama and colleagues observed that the bioavailability of EA complexed with a cyclodextrin, 30 min after a gavage (at the dose of 0.4 g/kg), was 7-fold higher if compared to the gavage of free EA [184]. Likewise, Mady and collaborators observed an enhancement of the water solubility (49.79 µg/mL vs. 9.73 µg/mL) and a 3-fold bioavailability improvement [185]. Currently, complexing with cyclodextrins appears as one of the best techniques to improve solubility and bioavailability, although the size of the substance seems to be a critical parameter to entry into the interior cavity.

Actually, another biopharmaceutical strategy is represented by the reduction of the particle sizes. Following the Noyes and Whitney equation, as the specific surface increases, the saturation solubility is enhanced, along with the capability to cross the intestinal barrier. Based on the particle size, it is possible to obtain micronized particles (if the size is less than 1 mm) or nanoparticles (if the size is less than 1 μm). Several authors tried to prepare micronized EA, observing a 6.5-times higher solubility than free EA, and even more interestingly, a 2-fold higher oral bioavailability [186]. Similarly, Mady and Shaker showed that the in vivo bioavailability of EA could be significantly raised when EA was administered in nanoparticles with poly-capro-lactone [187].

However, microparticles tend to agglomerate, resulting in a decrease of surface available for dissolution, whereas nanoparticles are very cohesive, and the tendency to aggregate is very strong; therefore, hydrophilic polymers or surfactants need to be used. In this respect, HC processes, introduced in Section 3.3, could be exploited in the extraction of pomegranate resources, as extremely efficient methods for particle size reduction and increase of particle dispersion. Applications for a few different foodstuff showing remarkable particle size reduction, such as tomato juice, milk protein concentrate and orange waste peel, were reviewed in Chapters 7 and 8 of a recent review [188]. A rotor-stator HC device used to treat wastewater effluents showed a four-fold decrease of particle size and a remarkable increase of specific surface area [189]. A fixed Venturi-shaped HC reactor applied to the treatment of biochar showed an enhancement of specific surface area by as much as 120%, due to the increase in meso- and micro-porosity, successfully exceeding conventional thermal methods [190].

In an application to pectin-rich biopolymers extracted from citrus waste, HC processes, implemented by means of a hydrodynamic rotational cavitation processor, were able to reduce by half the particle size in only 180 s, while increasing the dispersibility and stability of citrus pectin without affecting its composition, and exceeding the application of acoustic, i.e., ultrasonic, cavitation [191]. However, the effectivity of HC processes themselves were negatively affected by excessive pectin dispersion, suggesting that the processes should be held as short as possible.

Similar results were found in a comprehensive review of cavitation-based technologies for pretreatment and processing of food wastes, mainly aimed at the valorization of wastes into energy products [192].

Recalling the above-mentioned advantageous use of cellulose derivatives to obtain amorphous solid dispersions with EA, it is worthy of mentioning that the finding of semi-amorphous micronized cellulose as a by-product of the HC-based extraction of citrus waste peel [193,194]. As similar by-products can be expected from the HC-based extraction of whole pomegranate or PP, the resulting insoluble cellulose, possibly purified from lignin residues, could be used in the encapsulation of bioactive compounds released in the aqueous phase from pomegranate resources, adding to the efficiency and circularity of the processing chain.

Briefly, HC methods show promising application to the effective and efficient extraction of pomegranate resources in water only, aimed at providing bioactive compounds, such as ellagitannins and EA, in small and disperse particles, possibly along with micronized semi-amorphous cellulose as a by-product of the process and in principle applicable to the effective encapsulation of the bioactive compounds.

Finally, lipid-based formulations have been also studied. In particular, self-nano-emulsifying drug delivery system was carried out to improve solubility and absorption of EA. The formulation consisting of polyethylenglycol (PEG), polysorbate and caprylic/capric triacylglycerol allowed obtaining vesicle sizes of about 100 nm and an aqueous solubility definitely better than the free EA [195]. In agreement with this evidence, self-micro-emulsifying supersaturated, consisting of ethyl-oleate *plus* tween80 *plus* PEG *plus* polyvinylpyrrolidone K30, exhibited a more rapid release of EA and improved solubility [196].

## 6. Conclusions

*P. granatum* L., or pomegranate, is one of the most valuable medicinal plants in the pharmacological, industrial and commercial fields. To date, few products have been obtained from edible and inedible parts of the plant, including beverages, juices, jam, salad-based food, and additive in food coloring, cosmetic products including shampoo and bathing soaps, as well as ingredients for traditional medicine products and food supplements available on the market.

A few therapeutic fields in which pomegranate or its bioactive constituents might be used are also emerging; however, further research on the pharmacokinetics, the mechanisms of action and the standardization and properties enhancement of the extracts is required before drugs could be developed.

Although numerous formulation strategies have been tried to improve the water solubility and oral bioavailability of the most representative ellagitannins, this field remains to be adequately explored in order to fully exploit this valuable nutraceutical. Two main directions were identified. The first includes the complexation or encapsulation of pomegranate extracts with either metal nanoparticles or biopolymers, on which both research and industry have focused, resulting in advanced food supplements already available on the market. The second direction envisages the application of suitable green and scalable extraction techniques, bringing at the same time the advantage of allowing the affordable valorization of the huge amount of waste and by-products from pomegranate industrial processing, as well as the enhancement of the respective extracts.

Among such enabling techniques, which received far less attention, hydrodynamic cavitation appears extremely promising, although not yet exploited for pomegranate resources, with the potential to show comparatively higher extraction rates of bioactive compounds and process yields, as well as spontaneous complexation of ellagitannins, gallotannins and other polyphenols with pectin, as already found with the extraction of citrus fruit waste products, and furthermore, the chance to use the residues of extraction processes in the form of micronized cellulose as a further convenient encapsulation material.

The evidence suggests an extremely high potential to develop more effective and likely more affordable pomegranate extracts, rich in powerful bioactive constituents, as dietary supplements or adjuvants in therapies of cardiovascular and non-cardiovascular diseases.

## Data Availability

No new data were created or analyzed in this study. Data sharing is not applicable to this article.
